# Systems Biology as a Comparative Approach to Understand Complex Gene Expression in Neurological Diseases

**DOI:** 10.3390/bs3020253

**Published:** 2013-05-21

**Authors:** Leticia Diaz-Beltran, Carlos Cano, Dennis P. Wall, Francisco J. Esteban

**Affiliations:** 1 Systems Biology Unit, Department of Experimental Biology, University of Jaen, Campus Las Lagunillas s/n, Jaen, 23071, Spain; E-Mail: ldiazbel@ujaen.es; 2 Department of Computer Science, University of Granada, Daniel Saucedo Aranda s/n, Granada, 18071, Spain; E-Mail: ccano@decsai.ugr.es; 3 Computational Biology Initiative, Harvard Medical School, 250 Longwood Ave, Boston, MA 02115, USA; E-Mail: dpwall@hms.harvard.edu

**Keywords:** systems biology, neurological diseases, gene expression, autism, autism spectrum disorders, network analysis, protein-protein interactions, translational bioinformatics, behavioral diagnosis

## Abstract

Systems biology interdisciplinary approaches have become an essential analytical tool that may yield novel and powerful insights about the nature of human health and disease. Complex disorders are known to be caused by the combination of genetic, environmental, immunological or neurological factors. Thus, to understand such disorders, it becomes necessary to address the study of this complexity from a novel perspective. Here, we present a review of integrative approaches that help to understand the underlying biological processes involved in the etiopathogenesis of neurological diseases, for example, those related to autism and autism spectrum disorders (ASD) endophenotypes. Furthermore, we highlight the role of systems biology in the discovery of new biomarkers or therapeutic targets in complex disorders, a key step in the development of personalized medicine, and we demonstrate the role of systems approaches in the design of classifiers that can shorten the time for behavioral diagnosis of autism.

## 1. Systems Biology, a New Way of Thinking

We can define an organism as an individual living system, which is able to react to stimuli, grow, develop, reproduce and preserve a stable structure over time. Organisms are made up of diverse elements that are controlled by a complex network of interactions; for example, cells are composed by genes, proteins, metabolites and more that are connected by intricate relations. The biological pathways or networks that link the different components of living systems are not static; therefore, they are continuously evolving to adapt to internal and environmental changes [1].

The classical biological perspective has addressed the study of living organisms by focusing on isolated components instead of on the complex system as a whole. Such approaches have been successful in the identification and characterization of most living organisms’ parts, providing large amounts of information and knowledge about these single entities. However, this perspective has not helped to clarify the mechanisms of interaction between components and has been unable to predict the effects of changes and alterations that may occur in single and multiple components upon the dynamics of the entire system.

Systems biology can be considered as a new field of study that aims to understand biology at the system level, entailing the functional analysis of the structure and dynamics of cells and organisms. Therefore, rather than focusing on the characteristics of the isolated components of biological elements, the discipline of systems biology puts the focus on the interactions between them. Although the definition of systems biology varies, most scientists agree in that it complements the classic reductionist approaches in biomedical research and represents one of the best strategies to understand the underlying complexity of living systems.

**Figure 1:**
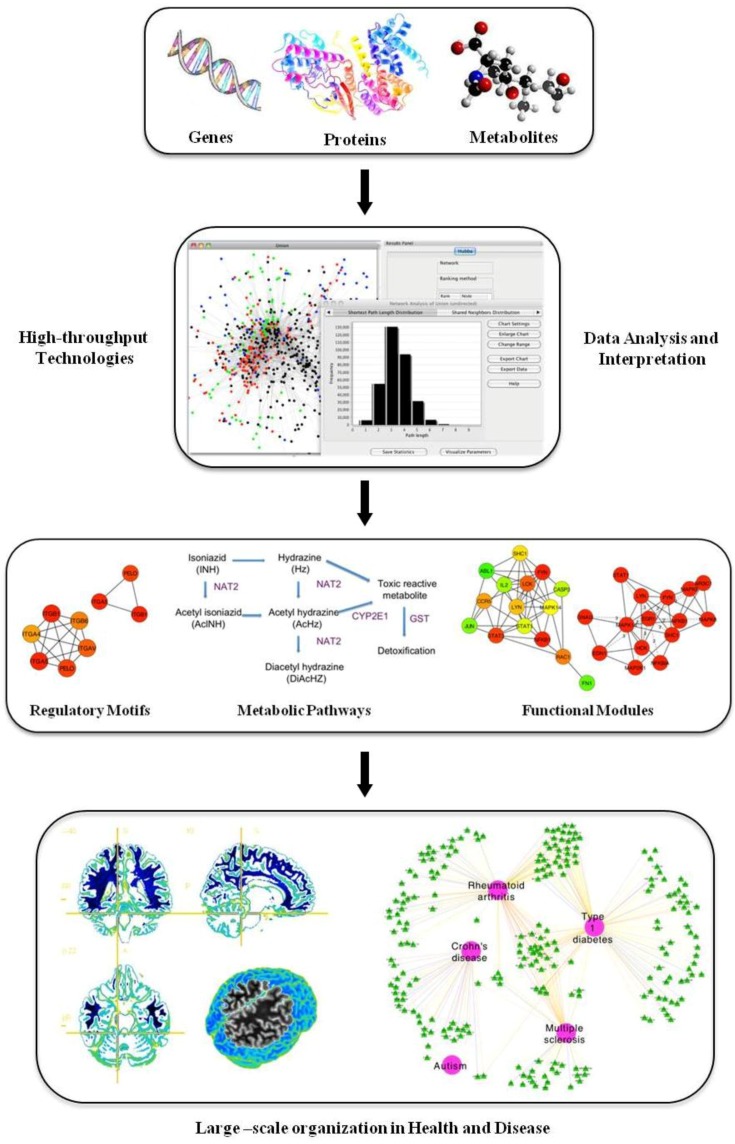
Scheme of a systems biology approach.

The basic principles of systems biology rely on the integration of multidimensional measurements through the use of multiple high throughput platforms, schemes and fields of study. At the simplest level, a systems approach focuses on the simultaneous examination of the whole system in contrast with traditional approaches that focus on a single gene, protein or metabolite. From a functional point of view, systems biology attempts to ascertain the biological pathways or networks that link the different components of a system and tries to identify the conditions that alter the equilibrium of these processes (Figure 1). Systems biology approaches applied to healthcare would try to identify the systems that, when altered, shift the body from a “healthy” to a “disease” state. The working hypothesis is that the elements of the biological system that are involved in the observed switch between states are specific to the disease and may be candidate targets for treatment to restore the system to its original healthy state [1].

We know that certain genetic changes alter gene expression, and, now decades of research exists that demonstrate how genetic variations bring about the molecular events that induce diseases and phenotypes.

All these accomplished efforts have produced the knowledge to populate many databases and generate web resources and tools for prioritization of genetic disease candidate genes. Our knowledge of the functional parts of the genome increases as databases of genome data keep growing, as well. A similar growth to that experienced by experimental approaches has been shown in the field of bioinformatics to comprehend the molecular results of genetic variations during the last years [2].

The development of high-throughput platforms (such as microarrays or sequencing technologies) represents an enormous challenge for researchers, because of the large amount of high-dimensional data generated. All this biological information must be analyzed from an integrative perspective that attempts to understand higher-level operating principles of biological processes. This is why systems biology has emerged to cope with this complexity and catalyze important changes in the future of healthcare. 

## 2. Systems Biology Approaches

The decade of the 90s represents the heyday of the genomic era. The advent of new technologies that lead to multiple experimental sources, such as microarrays, mass spectrometry and “omics”-data (genomic, proteomic, metabolomic, *etc.*), have meant a significant turnover for biological research and therapeutic targeting. From a biomedical standpoint, the translation of genetic discoveries into biological and therapeutic advances has changed dramatically since the completion of the Human Genome Project (HGP) in April 2003 [3]. The HGP represents an astonishing advance in molecular biology, as it gave us the ability to decipher our entire genome. Now, we have the power to analyze the polymorphisms of the approximately 30,000 genes for each individual and calculate probabilities about their disease likelihood.

Many disorders are known to be multifactorial diseases that are greatly influenced not only by genetic variants, but also by environmental signals. Therefore, it is equally important to measure the effects of these pathogenic factors. Taking into account that systems biology is the global analysis of the relationships established among the different constituents of a system in response to genetic or environmental changes, it is not strange that this discipline has arisen as a new approach to understand the behavior and the emergent properties of a system. Our system may be a few proteins carrying out a specific task or other type of molecules collaborating to accomplish a primal cellular or organic function.

The widespread emersion of systems biology has taken place by the crossover of four enabling facts: (1) the enormous amount of genetic information derived from the Human Genome Project (assembled and available gene, protein and metabolite data repositories); (2) the up-rise of interdisciplinary research efforts to create new technologies and integrative computational methodologies to better understand biological systems; (3) the development of high-throughput platforms for the integration of “omics” datasets to facilitate the detection, identification and assessment of molecular variability; (4) the rising of internetworking, which offers a powerful channel for data acquirement and the spreading of knowledge [4].

Taken together, the increase in systems biology applications is due to the advances developed in technology and experimental techniques (microarrays, mass spectrometry, computational approaches and web-related search engines and databases), which permit the simultaneous query of many components of a system. Systems biology can be regarded as a research tool that uses biological, chemical, statistical, physical, mathematical and computational methods to integrate and analyze molecular, physiological and clinical information extracted from laboratory experiments. As systems biology attempts to provide a comprehensive interpretation of all this knowledge, mutual high-throughput platforms for genomics, proteomics and metabolomics have been successfully created for the analysis, display and recording of information to guaranteed compatibility and accessibility to these data sets. 

Systems biology could be considered as a hypothesis-driven approach, in terms that we always begin with a descriptive, graphical or mathematical model, which is tested with suppositions that involve certain system changes, and the assembly of dynamic sets of data. Disparate data are merged and contrasted against the model; at each round of the process, the model is redeveloped. This procedure will follow, until the experimental data and the model are juxtaposed [4].

From a medical point of view, systems biology provides a primeval contribution to conduct functional analyses of genomic events that could be widely used in gene finding, biomarker identification, disease classification, drug discovery, therapy strategies and, in the last instance, predictive and preventive medicine. That is why systems biology is supposed to have had a great impact in biomedicine, as it is considered a highly productive tool, which provides valuable knowledge that should be applied in this field without any further delay. Thus, it becomes worthy to underline the relevance of primary research utilizing systems biology to comprehend both healthy biological systems and diseased states, because this knowledge will be fundamental to link possible biological failures to their corresponding disorders in order to anticipate and avoid disease.

Among other approaches, network analysis and functional annotation tools represent the best strategy for biomedical data interpretation. These integrative methodologies cope with higher levels of biological complexity, with the ultimate goal of understanding the underlying principles of intricate mechanisms in living systems and address the identification and prioritization of disease and drug candidate genes. In the next subsection, we are going to make a brief description of these systems biology approaches.

### 2.1. Network Analysis in Systems Biology

Discerning the causal agents of complex disorders becomes crucial for effective detection and for identifying the most adequate therapeutic interventions. Traditional approaches were pointed towards single molecules or signaling pathways when identifying diagnostic biomarkers. Instead, systems biology strategies focus on the global analysis of multiple interactions at different levels. A differentiated biological function is rarely regulated by a single molecule. Rather, the true nature of biological processes is far more complicated, and most biological features are determined by complex interactions among a cell’s distinct components. 

For this reason, systems biology strategies usually employ networks as a representation of these biological relationships, enabling one to take advantage of the mathematical tools from Graph Theory. Thus, groups of interacting molecules that regulate a discrete function construct biomodules whose interrelations bring out networks. In the network representation, nodes symbolize the constituents of the system (genes, proteins, enzymes) and links connecting nodes representing interactions or reactions in which these molecules participate [1]. 

Biological networks can be built by means of diverse approaches: (1) *de novo*, from direct experimental interactions; (2) by applying known interactions to an experimental -omic dataset or gene lists, either by hand or using specialized software, such as Ingenuity Pathway Analysis [5] and MetaCore [6], or known and predicted protein-protein interaction databases, such as String [7]; and (3) by reverse engineering. This last approach consists in gathering sufficient information to build a set of networks useful to predict the dynamics of the system in hand and to test the system behavior under several alterations in order to perform accurate network modeling simulations. 

**Figure 2:**
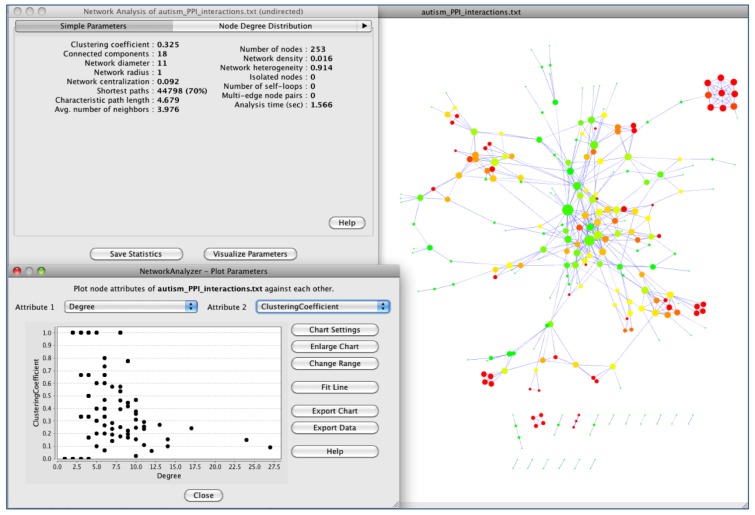
Topological analysis of autism protein-protein interaction (PPI) network using Cytoscape [8]. Genes associated with autism have been downloaded from Genotator (see section 4.2.), and PPI interactions have been obtained from String [7]. In autism PPI network, the large size of nodes represents a high degree, while dark colors mean values of clustering coefficients.

**Figure 3:**
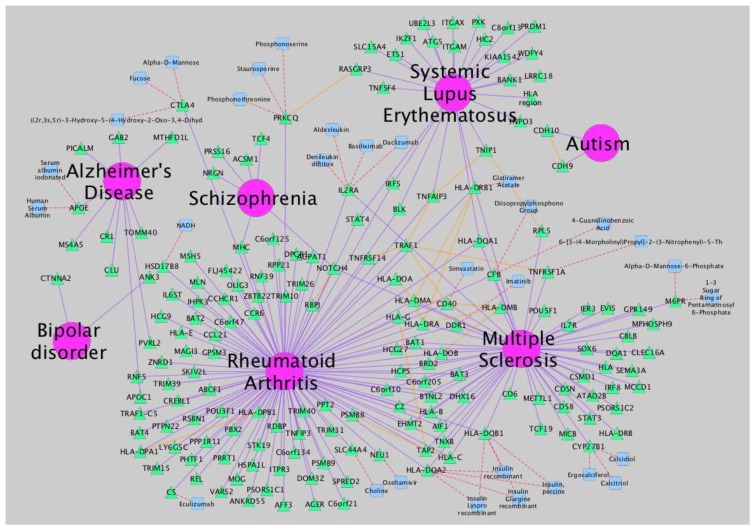
Network analysis of neurological disorders and related diseases using iCTNet [9]. Data to build the network is also provided by iCTNet. Several types of relationships (arcs) are represented: gene-disease association (purple), protein interaction among associated genes and select drug target interactions (red) and protein-protein interactions (orange). Node types are identified by shape and color: diseases (pink circle), genes (green triangle) and therapeutic drugs (blue square).

Complex network algorithms have been developed to visualize, analyze and model curated pathway datasets, integrate networks and perform functional annotations on them. In this field, Cytoscape [8] is a versatile, open-source software platform for complex network visualization and integration with any type of attribute data (Figure 2). Additionally, it incorporates several plugins that perform advanced topological analyses, modeling and data integration from different sources. In this sense, a recently developed approach, called iCTNet [9], is worth mentioning, which analyzes genome-scale biological networks for human complex traits with up to five layers of omics information—phenotype-SNP association, protein-protein interaction, disease-tissue, tissue-gene and drug-gene relationships—allowing the identification of genetic similarities among more than 200 diseases and the design of novel therapeutic interventions (Figure 3). Further information about biological network analysis can be found in [10] and [11], as cited in [12]. 

At this point, it becomes essential to make a brief description of biological networks concepts and structure to better comprehend biological processes. We can mainly differentiate three types of interaction networks: (1) protein-protein interaction, (2) metabolic and (3) signaling and transcriptional regulatory networks. These disparate networks are interconnected, framing a “network of networks” that is in charge of the cell’s demeanor. These networks are characterized by inherent topological features that transfer emergent properties of biological importance and can be analyzed globally. Some of the topological characteristics that define a network are: degree or connectivity (the number of links per node), the degree distribution (probability of a node having a specific number of edges), the clustering coefficient (degree to which nodes within a network cluster together), shortest path length (minimal distance, in number of edges, required to connect two nodes), robustness, *etc.* Detailed terminology, concepts associated with network analysis and further information can be found in [13]. 

Topological properties of biological networks offer valuable information about the behavior of the global system under study. Since the scale free nature of biological networks was brought out [14], general features of different types of networks have been sketched and progressively applied across disparate fields. Roughly speaking, in scale-free networks, the majority of nodes have few connections, while a few nodes (called hubs) are highly connected. This property confers scale-free networks a high robustness and makes them less vulnerable to environmental perturbations. The translation of this feature into the underlying biological system implies the assignment of a fundamental role to the hubs within the system. Thus, many key genes, proteins, enzymes and other molecules have been classified as hubs in their corresponding biological networks.

In all likelihood, cellular processes are performed in an extremely modular behavior. Generally speaking, modularity refers to a group of physically or functionally related nodes that collaborate to accomplish a relatively different function. Thus, in a network context, a module, subgraph or cluster comes out as a highly interconnected group of nodes. Actually, most compounds in a cell are constituents of an intracellular complex with modular activity or participants in a functional module as a temporary regulated component of a different process. A network with clearly-defined clusters suggests that it is spatter with different groups of highly interconnected nodes, which facilitates the appearance of detaches functional modules.

Despite the fact that network analysis represents a very useful tool for understanding the demeanor of complex systems from a global perspective, it is important not to forget individual components as elementary units in cellular networks. Motifs are defined as structural interconnected patterns overrepresented in the network in comparison to a randomized version of the same network. Thus, motifs represent basic recurrent patterns of interrelations that characterize a given network and, therefore, are of biological importance. Taking into account that a molecular constituent of a given motif frequently interacts with nodes that are not taking part in the motif, the way in which disparate motifs tie together needs to be addressed. Thus, it is clear that the identification of highly interconnected nodes (modules), and highly repetitive patterns in a network (motifs) can lead to the recognition of topological and functional modules that allows one to correlate these topological entities with their probable functional role. Different approaches have been addressed to discover modules in several different types of networks, using either topological features of networks or topology and functional genomic data, as cited in [13]. 

The mathematical models employed to create biological networks may be also used to predict the behavior of the network under specific perturbations. Systems biology attempts to ascertain how these alterations may affect the stability and robustness of the system by modeling the interrelation among the distinct components. A decisive stage in this modeling is the way in which networks are built from raw data (transcriptomics, proteomics, metabolomics, *etc*.). Several mathematical approaches are employed to carry out this task: Pearson correlations, differential equations, Boolean network-based methods, probabilistic models, *etc.* However, this is a very challenging task, also from a systems biology perspective [1]. As already mentioned, the different types of networks should not be considered in isolation, since they work together as a whole system, assembling a structured hierarchical network. For this reason, an alteration in a protein-protein interaction not only affects the protein interaction network, but it may also alter the metabolic network, having both perturbations and an associated effect in the final phenotype. Unfortunately, interrelations among disparate networks are not well known yet, although systems biology promises a better comprehension of biological network as a whole. The fast development of high-throughput technologies and the increasing use of computer science methods in the life sciences enclose the key to unravel the underlying mechanisms that control the transition from health to disease and set the guidelines that systems biology research will surely follow.

### 2.2. From Network Structure to Functional Analysis

There are several functional annotation tools to assist in extracting meaningful knowledge captured by the biological datasets and candidate gene lists derived from network analysis. Here, we briefly mention some of the most widely employed: (a) DAVID bioinformatics resources [15,16] allow gene annotation enrichment analysis, functional annotation clustering and gene functional classification, providing functionally related groups of genes that help unravel the biological content gathered by high throughput technologies; (b) Gene Ontology [17], a significant bioinformatics initiative that standardizes the representation of genes and gene products by developing three organizing principles, describing them in terms of their associated biological processes, cellular components and biological functions in a species-independent manner across multiple databases; (c) Ingenuity Pathway Analysis [5], a web-based application for modeling, analyzing, understanding and accurately interpreting complex biological and chemical meaning from genomic data. 

## 3. Systems Biology and its Application to Unravel the Complexity of Neurological Diseases

Complex diseases, such as neurological disorders, exhibit a great variety of molecular interactions involving a complex interplay between polygenetic and environmental factors. Thus, the combination of environmental conditions and a genetic background or somatic mutations may act as a trigger to a pathological state, which may frequently be induced by these disparate primary agents, operating alone or in synergy.

Classical reductionist approaches have mainly pointed towards key genes and their related products when attempting to characterize the cause and development of neurological diseases, offering an incomplete overview of these complex disorders. Therefore, although there are several cellular and molecular studies that have provided important insights about the nature of these conditions, a comprehensive understanding of their etiopathogenesis is still missing. 

Conversely, a systems biology perspective implies an integrated study of the underlying cellular and molecular pathways that control the functional processes determinant to create a physiological or pathological state within cells and organisms. Thus, a systems biology approach seems to be the better strategy to unravel the biological complexity of these multifactorial diseases involving several pathogenic determinants.

In a traditional manner, neuroscientists have dealt with brain complexity following a reductionist perspective, studying the different anatomic regions of this organ and characterizing their respective cellular components and basic functions in isolation. Under this approach, if a biological parameter and the appearance of a specific disorder correlate in a positive way, this fact is interpreted as a huge success, although the entire pathogenic process may still continue mostly uncharacterized. Systems biology complements this classical perspective not only because it is focused on comprehending not just the behavior of the different elements of the biological system under study, but also the results of the interactions among them, as well as its relation with the environmental conditions. Even though research endeavors are still focused on finding gene variants related with intelligence, memory and social capabilities, neurologists admit that the information-processing skills and, in the last instance, brain demeanor, are caused by the dynamic interaction of intricate synapse networks. Neurodegenerative disorders display a various range of phenotypes, but partake of common characteristics, such as progressive reduced cell function and survival within the nervous system that culminate in neurological incapacity and, eventually, death. 

### 3.1. Systems Biology Approaches

We can distinguish two different systems biology methodological approaches to investigate the chain of events leading to the appearance of neurological disorders and its subsequent development. The first one, known as descriptive, points towards system-wide analysis of biomolecular alterations (transcripts, proteins, lipids and metabolites), continued by the identification of key players in signaling pathways and disease processes. The second, which is more integrative, deals with higher degrees of biological complexity, identifying key modules or networks with intricate topological features at disparate levels. By analyzing structural properties and the connectivity of molecular networks, we obtain valuable information regarding their dynamic behavior and, thus, the depiction of their healthy or diseased steady state. These integrated studies enhance the chances of therapeutic treatment to readjust network’s dynamics towards the non-pathological state.

**Figure 4:**
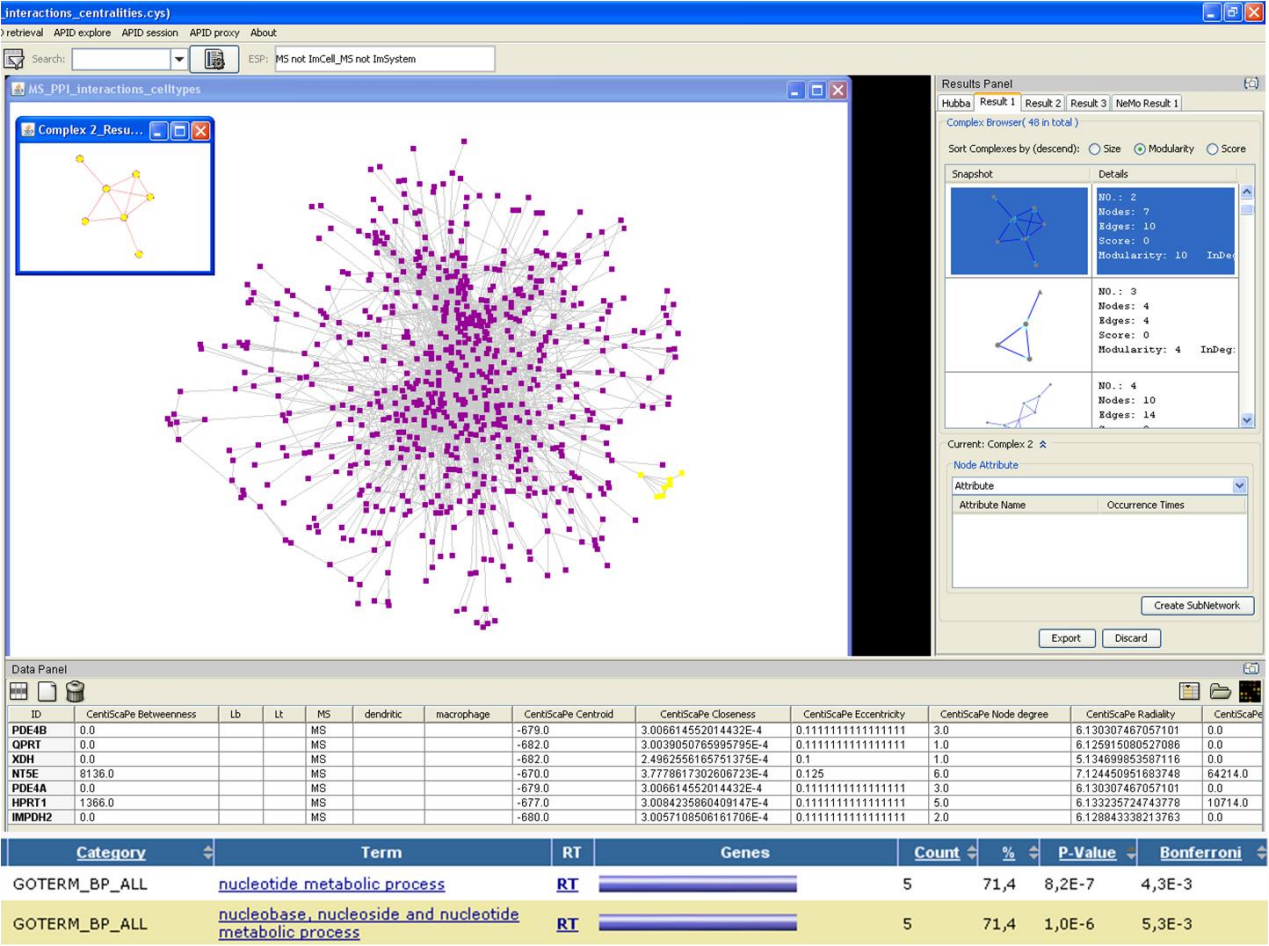
Search for modules in multiple sclerosis PPI network using Cytoscape [8]. At the bottom of the figure, we can see the result of the functional analysis of the module highlighted in yellow, conducted with DAVID [15,16].

As an example of how systems biology represents a discipline that may bring out important insights about the dynamics of neurological disorders, we are going to point out some advances that have been achieved using either descriptive or integrated systems biology approaches. Three neurodegenerative diseases (multiple sclerosis, Alzheimer’s disease and HIV associated dementia), reviewed elsewhere [18], were studied according to systems biology descriptive and integrative methods. Descriptive systems biology analyses showed common biological processes among the three disorders, such as inflammation, oxidative stress, altered microenvironment and programmed cell death. Although classical reductionist approaches had already pointed out these processes in neurological disorder pathogenesis, systems biology descriptive studies have revealed previously unidentified molecular determinant factors for each disorder. Nevertheless, the benefits of applying a systems biology perspective to the comprehension of neurological disorders may be increased by considering time as a key factor to study the evolution of these disorders pathogenesis. In this sense, samples taken in different stages of the disease in addition to samples taken from related disorders may identify important changes associated with the early stages of the disease. In a similar way, this scheme, along with blood or cerebrospinal fluid (CSF) serial analyses and together with pertinent animal models, might provide some temporal correlations, which may be important to sight a liable temporal resolution of the relevant pathogenic events. Experiments that take into account a temporal dimension offer a better basis for inferring and analysis of causality in comparison with studies conducted at a single time-point. Even though systems biology integrative approaches try to map the molecular interactions networks for living organisms, the studies reviewed for human neurological diseases mainly implicate the exploration of molecular modules or subnetworks (Figure 4). Successful examples of comparative network analysis in health and disease are considered the most hopeful branch of integrated studies. According to [18], modeling of biological networks dynamics, along with descriptive approaches that integrate a temporal dimension, represents the best strategy to comprehend, predict and change the disease course of neurodegenerative disorders.

### 3.2. Network-Based Studies

Another detailed review [19] highlights the utility of systems biology approaches, specifically network-based studies and pathway-centered analysis, for the understanding of the etiopathogenesis of neurodegenerative diseases. In this review, the authors emphasize the role of complex networks structural and dynamic analysis in the context of neurological disorders, pointing out that gene network-based studies of inheritable ataxias have revealed unknown pathways involved in RNA-splicing, which has been identified as a new pathogenic mechanism for these disorders. In this work, systems biology is outlined as a set of integrative approaches, which may accelerate drug discovery, by using computational models and validated cell assays to point out the best potential targets and dynamics subject to be altered, being also helpful for identifying new biomarkers, a key step in the contribution to the development of truly personalized medicine. New “omic” technologies and their application to systems biology offer new opportunities for biomarker discovery in complex disorders, as they integrate molecular data within models of disease pathogenesis, signaling pathways and biological networks [20]. This integration is vertically related across different levels of biological complexity (genes, molecules, cells, tissues and organisms) and to the clinical phenotype [21]. It is worth highlighting the role of quantitative network analysis as a useful approach that provides valuable information about the dynamics of the underlying pathogenetic events. As cited in [20], two strategies can be followed in order to achieve the integration of static data and system dynamics: analysis of network dynamics, using Bayesian inference, or Boolean networks and modeling, by using differential equations. 

#### 3.2.1. Bayesian Inference and Boolean Networks

The analysis of system dynamics is illustrated in several previous works [22], where Bayesian inference has been employed to identify (and later validate) a novel therapeutic target in multiple sclerosis (MS): Jagged-1. The authors combined Jagged-1 and IFN-beta as a therapy for this disease and performed a network simulation and analysis for this combined treatment. This analysis revealed gene interactions associated to the response to both treatments. Therefore, by comparing networks states before and after treatment with Jagged-1 and IFN-beta in patients with MS, the authors could recognize genes (including information about their clusters and interactions) and pathways related to the response to this therapy. Other works [23,24] have shown the power of Boolean networks to study signaling networks, from, which considerable quantitative information is already available. These works show how Boolean Networks join the complexity of network analysis with the simplicity of logic models and are able to provide a good connection between molecular interactions and cell phenotype. 

#### 3.2.2. Differential Equations

The second strategy, modeling by differential equations, takes advantage of the accuracy and power of mathematical models. However, obtained predictions are subjected to the availability of quantitative information, which is something very scarce for most of the biological processes that take place in complex diseases. Despite these constraints, several studies have developed models that recreate distinct aspects of the pathogenesis of MS and provide powerful insights for the discovery of biomarkers for this disease [25,26].

#### 3.2.3. The Importance of Network Parameters

Finally, it is worth commenting on an interesting study [27], where the authors report a novel approach to analyze protein-protein interaction networks at the meso-scale based on the products of genes differentially expressed in two degenerative diseases, multiple sclerosis and Alzheimer’s disease. In this work, the authors propose a novel perspective in their attempt to evaluate whether two essential parameters in network theory, degree and betweenness, are properties that show differences between implicated (seed proteins) and non-implicated nodes (neighbors) in these two diseases under study. Their findings showed that the degree of the seed proteins was lower than that of the neighbors, locating the first ones in peripheral regions of the network. According to the gene ontology analysis performed in the study, these peripheral regions are spread out among different pathways that may be implicated in the disease. Contrary to the topological involvements of the scale-free properties in biological networks, their results indicate that less-connected nodes are more suitable therapeutic targets in neurological disorders than hubs (highly-connected nodes). Therefore, the concept of multifactorial pathogenesis of neurodegenerative diseases becomes reinforced, since in this study, seed proteins are weakly-connected nodes participating in several pathways. Thus, if the desired goal were the modification of the disease course, it would be necessary to target many genes or proteins in distinct pathways. Several studies support these findings, as it is considered that hubs may not be good therapeutic targets, because of their key role in network modules; as we may know, biological networks would badly experience changes in hub demeanor without provoking relevant alterations across the network and, therefore, crucial side effects.

What can be gathered from the application of Systems Biology to the context of neurodegenerative diseases is that these approaches are making an important contribution to the identification of previously unrecognized molecular determinants for each disease and offer the possibility of novel therapeutic perspectives that will lead to a personalized medicine.

## 4. Application of Systems Biology in the Genome-Wide Search for Autism Gene Candidates

Autism is a complex neurodevelopmental disorder of early onset, manifested as a broad phenotypic range, the so-called autism spectrum disorders (ASD). Although it is clear that autism is a highly heritable disorder, causal molecular agents continue to be unknown, and it is still unclear whether the genetic part is a compounding of a few common variants or of many rare variants [28]. Researches that focus on single genes or mechanisms have been pushed into the background, as the complexity of autism gene space may only be discerned through the integration of this space into a fruitful set of hypothesis. At the present time, the exact etiopathogenesis of autism and ASD remains undefined, but it is likely to result from the combined effects of genetic, environmental, immunological and neurological factors. Due to the multifactorial nature of ASD, a systems biology perspective represents the best alternative to embrace the complexity of the biological processes and the enormous variety of molecular interactions that take place in ASD and its gene space. In the near future, new techniques for genotype clustering of ASD and the identification of their shared functional pathways could make it possible for us to find homogenous groups of autistic individuals, which could be combined for the purpose of elucidating their common underlying neurobiology [29].

Up to now, these endeavors have not revealed highly precise markers or validated therapeutic targets; for this reason, autism remains a behavioral diagnosis rather than a molecular one. Some of the behavioral symptoms of ASD include social anxiety and gaze avoidance, repetitive movements, hypersensitivity to touch, poor coordination, delayed speech and echolalia. In a very interesting manner, many of these manifestations are also exhibited in some neurological diseases, such as Tuberous Sclerosis, Hypotonia, Rett Syndrome and Fragile X Syndrome. These behavioral coincidences give rise to the assumption that there might be common molecular mechanisms, at least in part, among these diseases. 

### 4.1. Comparative Analyses

**Figure 5:**
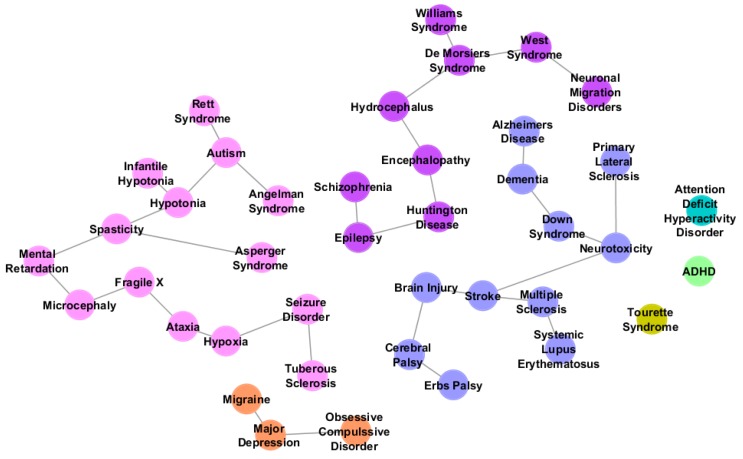
Neurological disorders related to autism listed by Autworks [35]. Different groups of colors represent disorder phylogeny. Nodes in pink refer to the autism sibling group, disorders that appeared to be most closely related to autism.

In this sense, some of us [30] conducted a large comparative analysis of autism and another 432 neurological diseases to describe a multi-disorder subcomponent of ASD. The hypothesis to validate was that diseases with behavioral similarities with autism might share many genes with this disorder. Undeciphering this genetic overlap may contribute to a better comprehension of the range of different manifestations associated with autism. To characterize the multi-disorder component of the autism network, gene lists of 433 neurological diseases listed by NINDS (National Institute of Neurological Disorders and Stroke) were generated utilizing GeneCards [31] and OMIM [32]. From these gene lists, a subset of diseases with three or more genes in common with autism was selected. The transformation of the gene lists into a presence-absence matrix made possible the generation of a disorder phylogeny that joined in the same group autism with 13 related disorders (autism sibling disorders), such as Microcephaly, Mental Retardation, Ataxia and Seizure Disorder (Figure 5). 

Using String [7], the authors generated gene networks for each member of the autism sibling group to find out genetic overlap with autism. The results showed that more than half of the published autism genes have been also associated to related neurological disorders. These findings prove that there is molecular overlap and indicate that these disorders might share molecular mechanisms with autism that could help to understand the genetic etiology of this complex disorder. The multi-disorder component of the autism network (MDAG) presented a high number of interconnections and biological process enrichment for synaptic transmission and central nervous system development.

In this study, two analytical strategies were devised to test whether the large extent of behavioral overlap between autism and its sibling disorders may provide significant guidance in the genome-wide search for autism gene candidates. The first, a process-based strategy, was based on the premise that processes for which the MDAG genes were enriched are mostly relevant for neurological dysfunction. In this sense, genes with implication in these processes, which have already been associated to one or more autism sibling disorders, but not to autism, are supposed to be autism gene candidates. To test this hypothesis, a gene expression dataset (GSE6575) from Gene Expression Omnibus (GEO) [33,34] was downloaded; this dataset consisted in 17 autistic patients with early onset and 12 healthy controls. The results obtained identified 154 genes not previously linked to autism, 42% of them were validated to be under significant differential expression in microarray data from autistic individuals. Taken together, the fact that these genes have been related to neurological dysfunction and implicated in some important autism processes make them high profile leads for a better comprehension of molecular pathology in autism. The second strategy, a network-based approach, was founded in the current mainstream understanding that protein-protein interaction networks may offer precious and often unexpected guidance for disease causing agents. In this study, instead of considering the entire protein interaction network, the authors filtered the group of interactions to MDAG genes, such that they enclosed only those proteins included in the list of sibling autism disorders, but missing from the list of published autism candidates. Through this approach, the authors uncovered 334 new genes that interact with published autism genes, 87% of them were found to be significantly differentially expressed in autistic individuals from the same microarray data when compared to healthy controls. 

Thus, in both analytical approaches, Wall *et al.* were able to use prior knowledge from biological processes and interaction networks to provide clusters of genes assumed to be differentially regulated in autism, demonstrating that the systems biology perspective appears as a valuable tool to acquire significant biological knowledge from genome-scale studies of complex disorders, such as ASD. 

### 4.2. Web-Driven Platforms

**Figure 6:**
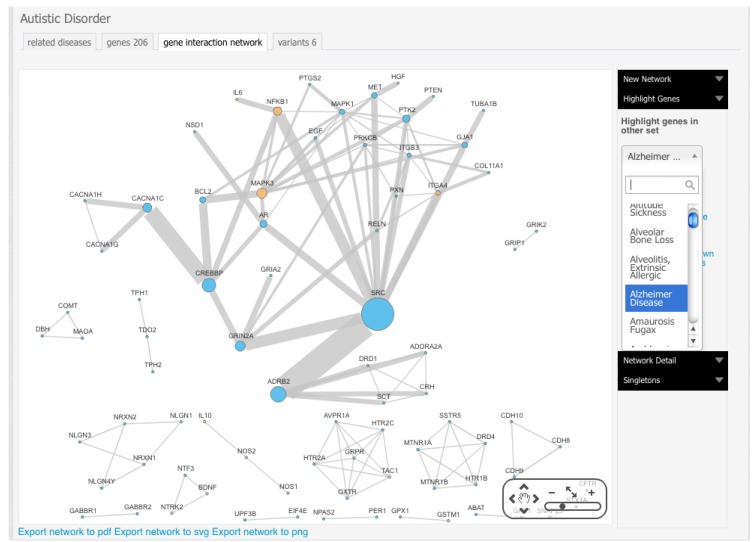
Cross-disease gene interaction comparison of Alzheimer’s disease with the autism network as provided by Autworks [35]. Orange nodes represent overlapped genes between these disorders.

With the ultimate goal of undeciphering the common molecular mechanisms that underlie the overlapping behaviors of autism with other neurological diseases, some of us have developed Autworks [35] (Figure 6), a web-driven navigation system that gives any researcher the opportunity to view and search through the networks of genes implicated in complex diseases and disorders, including autism. Autworks entails not only a way to see which genes have been linked to autism, but also to see how those genes interact with one another. The main hope is that by investigating the autism network and the complete set of interactions among all known autism genes, researchers will be able to find those gene combinations that cause the disorder and pave the way towards faster diagnosis and treatment. In this sense, if it is possible to systematically identify and characterize all similarities among a wide array of human disorders and diseases, we will be able to build a clear mapping between the genetics and the behaviors of autism, enabling us to literally redefine the disease and to find the genetic pieces that translate into real, clinically actionable tools for diagnosis and treatment. In addition, this constitutes a major initiative to break down communication barriers between research sites focusing on autism and individuals dealing with the disorder.

Motivated for an automated search of autism genes throughout different databases, some of us also launched Genotator [36], a source for comprehensive genetic annotation of disease, designed as a meta-query engine that provide high quality gene-disease associations based upon data from 11 highly reliable resources. The Genotator engine takes in a disease input and produces a ranked list of genes most likely to be associated with that disease. As an example, when using "autism" as the query term in the Genotator disease search, 701 genes were provided as a molecular signature of this disorder, as of the date hereof.

### 4.3. Microarray Games

It is worth highlighting an original systems biology approach that represents a more powerful method for valuable signal detection in autism gene expression microarray experiments. In a previous work [37], a mathematical analysis of the gene expression data based into the coalitional (or co-operative) game area of game theory was carried out. The aim was to prove whether this mathematical approach was able to identify candidate ASD genes from the analysis of a microarray experiment (Gene Expression Omnibus, GSE6575) [33,34] in which only a few genes could be obtained as differentially expressed, employing standard statistical approaches. The results showed that coalitional games enhanced in a significant manner the ability to detect ASD candidates, some of which have been involved in biological functions and disorders previously shown to be associated with ASD. Therefore, this novel data-driven approach to determine gene expression relevance from microarray experiments stands out as a useful methodology to decipher the expression demeanor of genes in the intricate molecular basis of ASD.

To conclude, this review just points out that, in the autism case, systems biology has also been employed as a technique to shorten behavioral evaluation and diagnosis processes of autism spectrum disorders. Current instruments for behavioral diagnosis of autism may be effective, but in the majority of cases, extremely expensive and time-consuming; up to now, the average age of diagnosis in the US is approximately six years, and this entails a significant delay in the delivery of appropriate behavioral therapy for a child’s development. As shown in [38], cutting-edge machine-learning methods may have an important role in speeding the pace of the initial evaluation of autistic individuals and broadening the range to a very much higher percentage of the population at risk. In this study, the authors performed a data-driven approach to select a reduced set of questions from the Autism Diagnostic Observation Schedule-Generic (ADOS) [39], one of the most widely used classifiers for autism diagnosis and assessment, which is composed of four modules, each customized for a specific group of individuals based on their level of social and communications behavior. Applying a series of machine-learning algorithms, they found and validated the ADTree classifier, consisting of just eight questions (72.4% less than the complete ADOS Module 1) and capable of distinguishing individuals with autism from individuals without autism with almost perfect sensitivity, specificity and accuracy. Most importantly, this classifier provides an earlier diagnosis of autism in a critical window of development in which behavioral therapy would have a significant impact on disease evolution and quality of life. Thus, these findings may prove valuable in the development of mobile health approaches for preliminary evaluation and clinical prioritization, such that families can be granted care earlier than under current diagnostic practices.

## 5. Conclusions

Systems biology emerges as an essential tool to manage the huge amount of information generated by the fast development of high-throughput research technologies and to increase the comprehension of biological processes that might be analyzed from a global perspective. These descriptive and integrative methodologies are providing a vital contribution to the understanding of the underlying biological pathways and processes that determine the etiopathogenesis of complex diseases, including autism. Although more advances are yet to be accomplished, the advent of systems biology represents an innovative research context and, therefore, a turning point, which is potentially capable of transforming the design and implementation of new drug discovery and development.
